# Trends of Diphtheria–Tetanus–Pertussis and Measles Vaccine Coverage Preceding and during the COVID-19 Pandemic: An Analysis of the WHO European Region from 2000 to 2022

**DOI:** 10.3390/vaccines12101145

**Published:** 2024-10-06

**Authors:** Andrea Maugeri, Martina Barchitta, Giorgia Cappuccio, Giuliana Favara, Roberta Magnano San Lio, Antonella Agodi

**Affiliations:** Department of Medical and Surgical Sciences and Advanced Technologies “GF Ingrassia”, University of Catania, 95123 Catania, Italy; andrea.maugeri@unict.it (A.M.); mbarchitta@unict.it (M.B.); giorgiacappuccio@gmail.com (G.C.); giuliana.favara@unict.it (G.F.); robertamagnanosanlio@unict.it (R.M.S.L.)

**Keywords:** pediatric vaccination, measles, DTP, COVID-19, pandemic

## Abstract

Available data highlights the significant impact of the COVID-19 pandemic on global vaccination trends. Despite this, comprehensive evaluations of these changes at the European level are still scarce. This study examines coverage for diphtheria, tetanus, and pertussis (DTP) and measles-containing vaccines (MCV) in the WHO European Region from 2000 to 2022. Vaccination coverage data, defined as the percentage of surviving infants who received the first and third doses of DTP (DTP1 and DTP3) and the first and second doses of MCV (MCV1 and MCV2), were extracted from UNICEF databases. Joinpoint regression analysis was employed to identify joinpoints in the time series and to estimate Annual Percent Changes (APCs) and Average Annual Percent Changes (AAPCs) over predefined timeframes. The coverages for DTP1 and MCV1 exhibit a similar trend, overall characterized by four joinpoints, one of which is in 2019. In contrast, the coverage for DTP3 does not show a significant temporal trend and lacks joinpoints, while the vaccination coverage for MCV2 shows a steadily increasing trend, with three identified joinpoints. A comparative analysis with the pre-pandemic period reveals a significant increase in the number of countries characterized by a decreasing trend during the pandemic period for all considered vaccination coverages, as indicated by the negative AAPC values. These results highlight the effect of the pandemic on childhood vaccination coverage. Compared to a mere descriptive analysis, the temporal analysis of trends using joinpoint regression provides significant opportunities to identify variations in vaccination coverages and pinpoint areas for intervention in future vaccination strategies.

## 1. Introduction

The consequences of the COVID-19 pandemic have been profound, leading to direct losses of millions of lives due to the virus. The resulting global impact in terms of morbidity, mortality, and societal disruption prompted governments worldwide to implement drastic measures such as population-wide lockdowns, border closures, the suspension of mass gatherings, and the World Health Organization (WHO) guidelines recommending a temporary halt to mass immunization programs globally [1]. While these measures aimed to control the outbreak, they have seriously threatened previous achievements in immunization services, significantly impacting global endeavors in eradicating and eliminating vaccine-preventable diseases (VPDs). In May 2020, the WHO reported that at least 80 million infants under one year of age were at risk of missing essential vaccinations [2]. With a sustained decline in vaccine coverage rates, there has been a noticeable increase in outbreaks of measles, diphtheria, pertussis, and other VPDs [3]. In EU/EEA countries, measles cases have sharply increased since 2023, with nearly 6000 cases and at least five deaths reported between March 2023 and February 2024 [4]. Pertussis cases have also spiked, showing a more than tenfold increase during the same period compared to previous years [4]. To address these outbreaks, it is crucial to close immunity gaps by identifying those who missed or delayed vaccinations. Routine immunizations and booster doses, recommended at different life stages, are essential, and individuals should regularly consult healthcare providers to ensure they are up to date with vaccinations.

The available data underscores the profound impact of the COVID-19 pandemic on vaccination trends worldwide. A study has revealed a significant global reduction in the administration of DTP3 (diphtheria, tetanus, and pertussis third dose) and MCV1 (measles-containing vaccine first dose) across various WHO regions during the initial half of 2020 as compared to the preceding year [5]. The majority of countries experienced their lowest vaccination numbers in the months of April and May 2020. However, the study also highlights proactive measures taken by many countries in the form of catch-up vaccination plans [5]. Furthermore, the study underscores the significance of *Immunisation Agenda 2030*, a pivotal document outlining strategies and challenges geared toward achieving optimal vaccination coverage [5]. Reports from the WHO and UNICEF highlight a significant decline in childhood vaccinations globally [2,6]. Between 2019 and 2021, there was a substantial 5% reduction in the proportion of children globally receiving DTP3, marking the most significant decline in the past three decades. The repercussions of this decline are substantial, with more than 25 million children worldwide missing one or more doses of DTP in 2021—this is an alarming increase of over 6 million compared to 2019 [2,6].

The accurate and systematic evaluation of these changes at both regional and global levels presents some challenges. More specifically, most studies have been conducted amid the ongoing COVID-19 pandemic, providing only a partial snapshot of the situation but revealing discernible trends [3,7,8,9,10,11,12]. Additionally, these studies have been predominantly carried out in African [9,13] or American countries [10,11,12]. The number of studies in Europe remains limited, with indications of reduced vaccine demand, decreased availability of healthcare workers, and challenges reported by specific countries regarding vaccine supply [3,7,8]. Nevertheless, the WHO European Region, consisting of 53 diverse countries, presents a unique setting to investigate the impact of the COVID-19 pandemic on vaccine coverage. This region encompasses various healthcare systems, socioeconomic conditions, and cultural factors that can influence immunization programs differently. Understanding the impact of the COVID-19 pandemic on the coverage of DTP and measles vaccines is crucial for assessing potential disruptions and identifying areas for intervention. Temporal analysis of vaccine coverage trends can provide valuable insights into the effect of the pandemic on vaccination rates and identify potential factors contributing to changes in coverage levels.

Therefore, this study aims to analyze the temporal patterns of DTP and MCV coverage in the WHO European Region from 2000 to 2022, specifically focusing on the periods preceding and during the COVID-19 pandemic. By examining trends in vaccine coverage over time, this analysis aims to pinpoint disruptions or changes in vaccination rates, contributing to the formulation of hypotheses about potential influencing factors.

## 2. Materials and Methods

Vaccination coverage data, defined as the percentage of surviving infants who received DTP1-3 and MCV1-2 vaccinations, were extracted from UNICEF databases covering the period from 2000 to 2022 [14]. At the regional level, descriptive statistics, including mean and standard deviation (SD), were used to summarize the information for the entire period. The annual change was initially evaluated in terms of relative change (%) by calculating the difference between the current year’s vaccination coverage and the previous year’s coverage, divided by the previous year’s coverage. At the country level, vaccination coverage data for 2022 were initially presented using a choropleth map. The descriptive analysis was conducted using IBM SPSS Statistics (version 26.0, IBM Corp., Armonk, NY, USA) and Excel 2022.

The joinpoint regression analysis was conducted using the Joinpoint Regression Program (version 4.9.0.0; Statistical Research and Applications Branch, National Cancer Institute, Bethesda, MD, USA). This analytical method assesses trends over time and is particularly useful for analyzing rates, proportions, or any time-dependent measure. The primary objectives are to identify potential time points where significant changes in trends occur (joinpoints) and to estimate the regression function with these joinpoints. What distinguishes joinpoint regression from other models, such as piecewise or segmented regressions, is its emphasis on continuity at the change-points. Unlike these models, joinpoint regression maintains smooth transitions between segments rather than abrupt transitions. Additionally, joinpoint regression automatically determines the number and placement of joinpoints within the model, rather than requiring a predetermined specification.

The process of determining joinpoints generally involves a grid search method, as recommended by Lerman [15]. This method entails creating a grid representing all potential positions for joinpoints and fitting the model for each combination. The optimal number and placement of joinpoints are then determined by minimizing the sum of squared errors (SSE). This selection process involves hypothesis testing, wherein simpler null models are compared against more complex alternative models until an optimal fit is achieved. The joinpoint regression analysis was applied on the logarithmically transformed outcome variable and assuming uncorrelated errors. The grid search method was applied to determine the optimal number of joinpoints, from a minimum of 0 to a maximum of 5. Model selection was based on a permutation test (5000 permutations) with a significance level of 0.05. To account for multiple comparisons, an approximate permutation Monte Carlo method was employed to calculate *p*-values under the null hypothesis. Additionally, the Bonferroni adjustment was applied to control the overall type I error rate. The analysis generated Annual Percent Changes (APCs) and their 95% Confidence Intervals (95% CIs), indicating the average yearly percentage change between identified joinpoints. Additionally, Average Annual Percent Changes (AAPCs) were calculated as an aggregated measure of trends over specified timeframes. This metric represents the mean APCs across several years, accounting for any shifts identified by the joinpoint model. The AAPC is computed as a weighted average of the APCs, with weights based on the duration of each APC interval. Specifically, a comparative analysis was performed between the pre-pandemic period (2000–2019) and the pandemic period (2019–2022) to evaluate variations in AAPCs.

## 3. Results

### 3.1. Vaccination Coverage in the European WHO Region

This study analyzed vaccination coverages for DTP1 and 3 and MCV1 and 2 from 2000 to 2022 in the WHO European Region.

**Figure 1:**
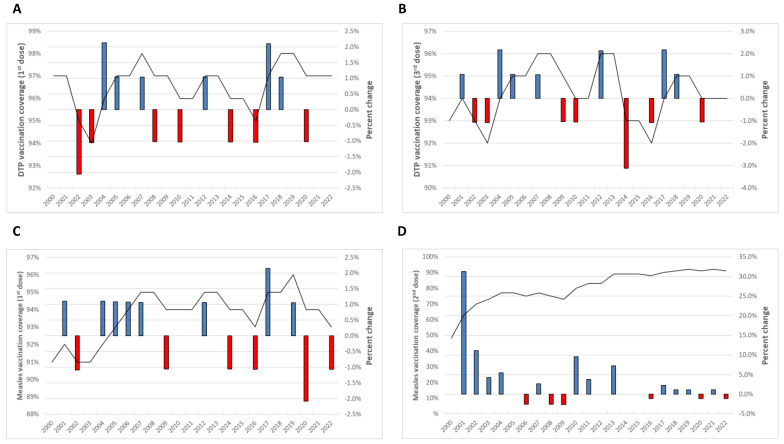
Annual vaccination coverage and yearly relative changes in the WHO European Region from 2000 to 2022 for DTP1 (**A**), DTP3 (**B**), MCV1 (**C**), and MCV2 (**D**).

Between 2000 and 2022, the average percentage of surviving infants who received DTP1 in Europe was 96.6% (SD = 1.0%) (Figure 1A). The lowest value, 94%, occurred in 2003, while peaks of 98% were noted in 2007, 2018, and 2019. In 2022, the vaccination coverage for DTP1 was 97%, and no overall change was observed since 2000. Annually, the most significant increase occurred at 2.1% from 2003 to 2004 and 2016 to 2017, whereas the most substantial decrease was −2.1% from 2001 to 2002. Notably, amidst the COVID-19 pandemic, vaccination coverage decreased from 98% in 2019 to 97% in 2020.

From 2000 to 2022, the average percentage of surviving infants receiving DTP3 stood at 94.2% (SD = 1.2%) (Figure 1B). The minimum value was 92% in 2003 and 2016, while the maximum was in 2007, 2008, 2012, and 2013, reaching 96%. A slight change of 1.1% was noted when comparing vaccination coverage between 2000 (93%) and 2022 (94%). The most significant annual increases, at 2.2%, occurred from 2003 to 2004 and again from 2016 to 2017. The largest decrease was observed between 2013 and 2014, with a decline of 3.1%. During the two years of 2019–2020, a reduction was observed from 95% in 2019 to 94% in 2020.

Examining MCV, particularly the percentage of surviving infants who received MCV1, reveals an average vaccination coverage of 93.65% between 2000 and 2022 (SD = 1.4%) (Figure 1C). The minimum value was 91% in 2000, 2002, and 2003, while the maximum was 96% in 2019. In 2022, the vaccination coverage stood at 93%. The maximum annual increase of 2.2% occurred from 2016 to 2017, while the maximum decrease of −2.1% was reported from 2019 to 2020. This period also coincides with the impact of the COVID-19 pandemic that we are examining.

Regarding the percentage of children who received MCV2, an average of 80.7% was observed (SD = 11.0%) (Figure 1D). The minimum value was 48% in 2000, while the maximum was 92% in 2019 and 2021. In 2022, the vaccination coverage reached 91%, marking a notable increase of 89.6% compared to the levels in 2000. On an annual basis, the maximum increase was 31.3% from 2000 to 2001, while the maximum decrease was −2.7% from 2008 to 2009. A reduction was observed from 92% in 2019 to 91% in 2020.

### 3.2. Vaccination Coverage in European Countries

**Figure 2:**
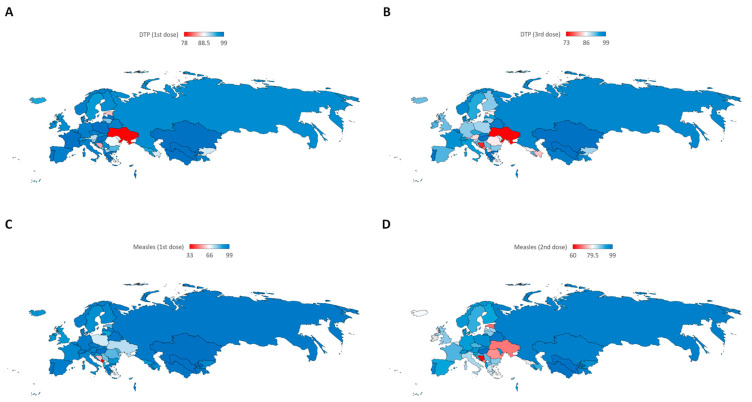
Country-level vaccination coverage in 2022 for DTP1 (**A**), DTP3 (**B**), MCV1 (**C**), and MCV2 (**D**).

The study also examined vaccination coverages for DTP and MCV across countries within the WHO European Region. Figure 2 presents maps illustrating vaccination coverages in 2022.

Concerning DTP1, in 2022, the minimum coverage was 78% in Ukraine, followed by 85% in Bosnia and Herzegovina and 86% in Estonia. The maximum coverage reached 99% in 12 countries including Andorra, Greece, Hungary, Israel, Kazakhstan, Latvia, Luxembourg, Malta, Monaco, Poland, Portugal, Turkmenistan, and Uzbekistan. In 2000, the minimum coverage for DTP1 was 83% in Azerbaijan, followed by 85% in Tajikistan and 89% in Georgia. The maximum coverage of 99% was recorded in 21 countries (Andorra, Austria, Belarus, Czechia, Finland, France, Hungary, Iceland, Kyrgyzstan, Latvia, Lithuania, Luxembourg, Monaco, Netherlands, Norway, Poland, Portugal, Romania, Slovakia, Sweden, Ukraine, and Uzbekistan).

In 2022, the minimum coverage for DTP3 was 73% in Ukraine, followed by 75% in Bosnia and Herzegovina and 80% in Montenegro. The maximum coverage was 99% in seven countries (Greece, Hungary, Kazakhstan, Luxembourg, Monaco, Portugal, and Uzbekistan). In 2000, the minimum coverage was 76% in Azerbaijan, followed by 80% in Georgia and 81% in Austria. The maximum coverage was 99% in 17 countries (Belarus, Finland, Hungary, Kyrgyzstan, Latvia, Lithuania, Luxembourg, Monaco, Netherlands, Norway, Poland, Portugal, Romania, Slovakia, Sweden, Ukraine, and Uzbekistan).

Regarding MCV1, the minimum coverage in 2022 stood at 33% in Montenegro, followed by 58% in Bosnia and Herzegovina, and 71% in North Macedonia and Poland. The maximum value reached 99% in five countries (Hungary, Israel, Kazakhstan, Luxembourg, and Uzbekistan). In 2000, the minimum coverage was 67% in Azerbaijan, followed by 73% in Georgia and 74% in Italy and Malta. The maximum coverage was 99% in six countries (Denmark, Hungary, Kazakhstan, San Marino, Ukraine, and Uzbekistan).

In 2022, MCV2 exhibited its lowest coverage in Bosnia and Herzegovina (60%), followed by 68% in Estonia and 69% in Ukraine. The highest coverage reached 99% in Hungary and Uzbekistan. In 2000, MCV2 had a minimum coverage of 35% in Austria, followed by 48% in Portugal and 59% in Tajikistan. The maximum coverage was 99% in Hungary and Slovakia. Notably, Hungary and Uzbekistan achieved a maximum coverage of 99% for all four vaccines in 2022. This distinction was held by Hungary and Ukraine in 2000. In particular, Ukraine experienced a significant decrease of −21.2% in vaccination coverage for DTP1 from 2000 to 2022. Similarly, notable decreases were observed in Estonia (−10.4%) and Romania (−10.1%). In contrast, an increase of 11.4% was found in Tajikistan, followed by 8.4% in Azerbaijan and 6.7% in Georgia. A comparable trend was noted for DTP3, with Ukraine reporting a decline of −26.3% from 2000 to 2022, followed by decreases of −14.1% in Romania and −11.8% in Bosnia and Herzegovina. Conversely, the most significant increase was observed in Tajikistan (16.9%), followed by Greece (11.2%) and Azerbaijan (9.2%). Regarding MCV1, significant decreases from 2000 to 2022 were evident in Bosnia and Herzegovina (−27.5%), North Macedonia and Poland (−26.8%), and Ukraine (−25.3%). The highest increase was recorded at 38.8% in Azerbaijan, followed by 29.7% in Malta and 27.0% in Italy. Finally, MCV2 decreased by −30.3% in Ukraine, 26.0% in Romania, and 23.6% in Estonia. The maximum increase was seen in Austria with a percentage of 168.6%, followed by 100.0% in Portugal and 64.4% in Tajikistan.

### 3.3. The Potential Impact of COVID-19 Pandemic on Vaccination Coverage

**Figure 3:**
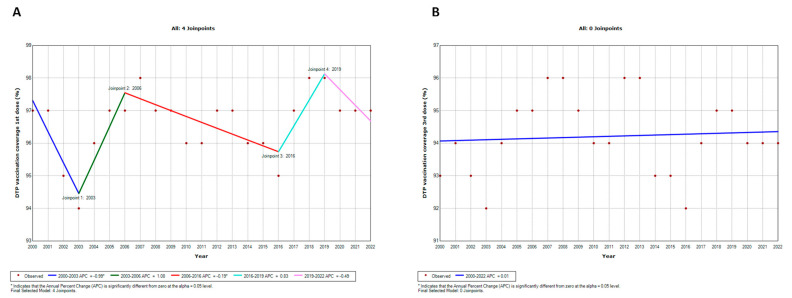
Joinpoint regression analysis of DTP1 (**A**) and DTP3 (**B**) coverage in the WHO European Region from 2000 to 2022.

**Figure 4:**
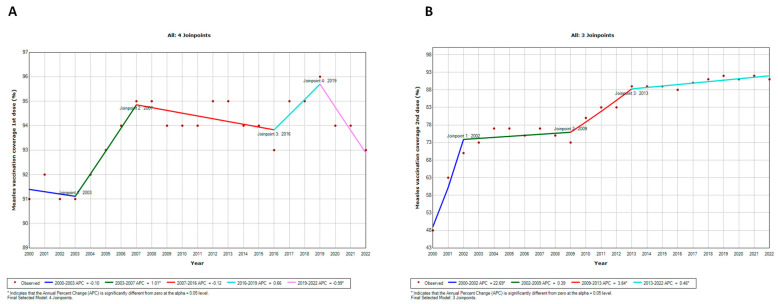
Joinpoint regression analysis of MCV1 (**A**) and MCV2 (**B**) coverage in the WHO European Region from 2000 to 2022.

To assess the potential impact of the COVID-19 pandemic, our initial step involved examining the temporal trends in vaccination coverage in the WHO European Region using joinpoint regression analysis (Figure 3 and Figure 4).

Regarding DTP, the analysis identified four joinpoints for the first dose (DTP1; Figure 3A) and none for the third dose (DTP3; Figure 3B). Specifically, vaccination coverage for DTP1 declined annually from 2000 to 2003 (APC = −0.99; CI = −1.93; −0.03), then increased until 2006 (APC = 1.08; CI = −0.84; 3.03). Over the subsequent years (2006–2016), there was a slight but significant decrease (APC = −0.19; CI = −0.36; −0.01), followed by an increase until 2019 (APC = 0.83; CI = −1.08; 2.77). However, vaccination coverage subsequently experienced a decline (APC = −0.49; CI = −1.44; 0.46).

Vaccination coverage for MCV1 followed a trend similar to DTP1, with four joinpoints identified (Figure 4A). Specifically, it decreased annually from 2000 to 2003 (APC = −0.10; CI = −1.03; 0.83), then rose until 2007 (APC = 1.01; CI = 0.08; 1.95). Subsequently, from 2007 to 2016, there was a slight decrease (APC = −0.12; CI = −0.32; 0.08), succeeded by an increase until 2019 (APC = 0.66; CI = −1.19; 2.54). However, vaccination coverage then declined (APC = −0.99; CI = −1.91; −0.07). Instead, vaccination coverage for MCV2 exhibited a continuously increasing temporal trend, characterized by three joinpoints (Figure 4B). From 2000 to 2002, there was a substantial increase (APC = 22.69; CI = 16.75; 28.92), followed by an almost stable phase until 2009 (APC = 0.39; CI = −0.45; 1.23). From that point, the increase became significant again until 2013 (APC = 3.84; CI = 1.20; 6.44), before slowing down (APC = 0.46; CI = 0.01; 0.91).

### 3.4. Stratified Analyses at the Country Level

**Figure 5:**
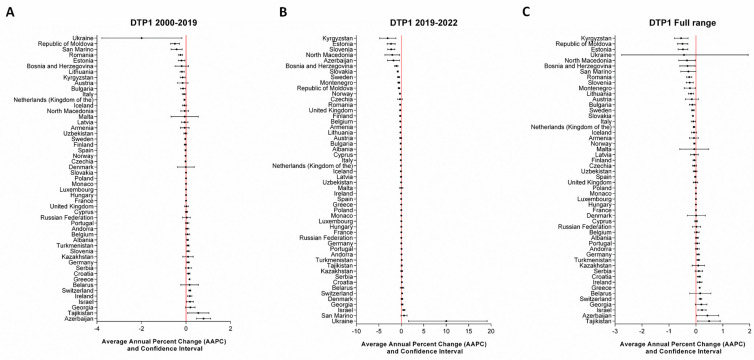
AAPCs of DTP1 vaccination coverage at the country level across predefined timeframes.

**Figure 6:**
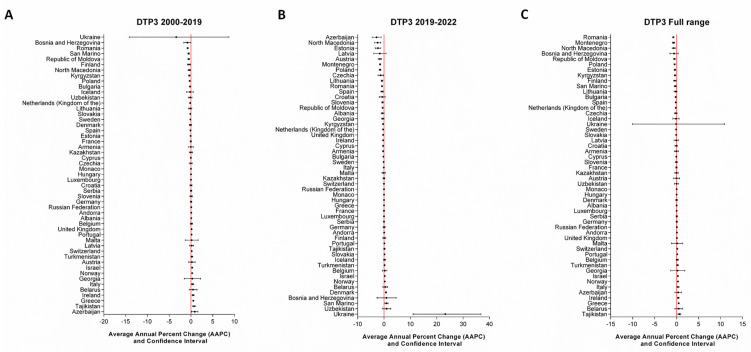
AAPCs of DTP3 vaccination coverage at the country level across predefined timeframes.

**Figure 7:**
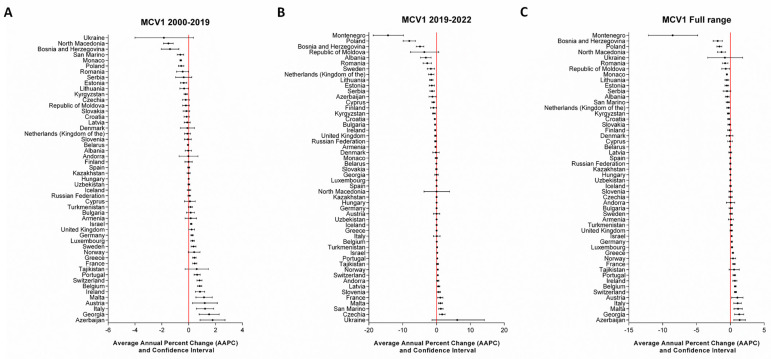
AAPCs of MCV1 vaccination coverage at the country level across predefined timeframes.

**Figure 8:**
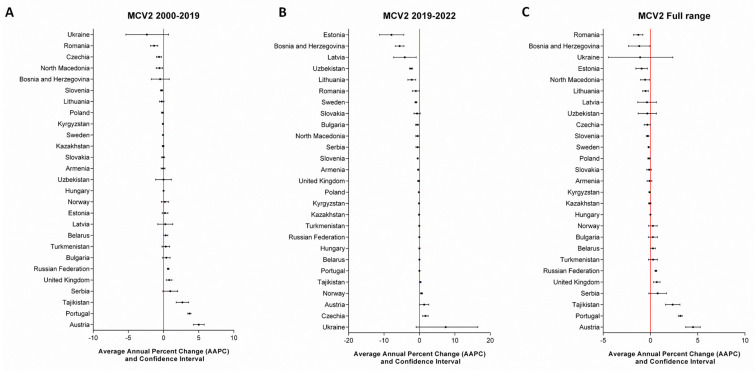
AAPCs of MCV2 vaccination coverage at the country level across predefined timeframes.

At the national level, a joinpoint in 2019, preceding the onset of the COVID-19 pandemic, was identified for specific countries: Kyrgyzstan and Slovakia for DTP1; Czechia and Latvia for DTP3; Albania, Czechia, Finland, Republic of Moldova, and Romania for MCV1; and Latvia and Lithuania for MCV2. The number of joinpoints identified for each country, as well as the APC for each time period, are reported in the Supplementary Materials. To comprehensively assess the potential impact of the pandemic at the country level, we also examined the temporal trends in vaccination coverage across fixed time periods (2000–2019, 2019–2022, and the full range from 2000 to 2022). The AAPC values are presented visually in Figure 5, Figure 6, Figure 7 and Figure 8 and fully detailed in the Supplementary Materials.

For DTP1 vaccination coverage during the 2000–2019 period (Figure 5A), 13 out of 51 countries experienced a significant decrease (AAPC and its confidence interval < 0), while 11 out of 51 countries saw a significant increase (AAPC > 0). However, during the pandemic period (2019–2022) (Figure 5B), the number of countries with a significant decreasing trend increased to 22 out of 52, with AAPC ranging from −0.01 (CI = −0.01; −0.01) in Greece to −3.02 (CI = −4.81; −1.19) in Kyrgyzstan. Only 5 out of 52 countries reported a significant increase, notably with Ukraine reporting the highest value (AAPC = 10.02; CI = 1.62; 19.11). Across the full range, 16 out of 52 reported significantly negative AAPC values, while 9 out of 52 had positive values (Figure 5C).

For DTP3 vaccination coverage during the 2000–2019 period (Figure 6A), 12 out of 51 countries experienced a significant decrease, while 11 out of 51 countries saw a significant increase. Similarly, the number of countries with a significant decreasing trend increased during the 2019–2022 period (20/52), with AAPC values ranging from −0.12 (CI = −0.17; −0.06) in Sweden to −2.82 (CI = −4.52; −1.08) in Azerbaijan. Only 6 out of 52 countries reported a significant increase; once again, Ukraine reported the highest value (AAPC = 23.23; CI = 11.04; 36.76) (Figure 6B). Across the entire range, 14 out of 52 reported significantly negative AAPC values, while 8 out of 52 had positive values (Figure 6C).

For MCV1 vaccination coverage during the 2000–2019 period (Figure 7A), 10 out of 51 countries experienced a significant decrease, while 16 out of 51 countries saw a significant increase. However, the number of countries exhibiting a significant decrease rose during 2019–2022 (21/52), with AAPC values ranging from −0.05 (CI = −0.1; −0.01) in Belarus to −14.35 (CI = −18.69; −9.79) in Montenegro. On the other hand, eleven countries showed an increasing trend (Figure 7B). Across the entire range, 16 out of 52 reported significantly negative AAPC values, while 15 out of 52 had positive values (Figure 7C).

For MCV2 vaccination coverage during the 2000–2019 period (Figure 8A), 7 out of 27 countries experienced a significant decrease, while 6 out of 27 countries saw a significant increase. The number of countries with a significant decrease rose during 2019–2022 (12/27), with AAPC values ranging from −0.15 (CI = −0.28; −0.02) in Poland to −7.88 (CI = −11.23; −4.4) in Estonia. On the other hand, four countries showed an increasing trend (Figure 8B). Across the entire range, 9 out of 27 reported significantly negative AAPC values, while 6 out of 27 had positive values (Figure 8C).

## 4. Discussion

The worldwide repercussions of the COVID-19 pandemic have been significant, resulting in the direct loss of millions of lives attributed to the virus. One potential outcome of the pandemic has been a decline in vaccination rates. This decline can be attributed to several factors, including disruptions to healthcare services, fear of exposure to the virus at vaccination sites, and misinformation leading to vaccine hesitancy. As a result, many individuals have postponed or foregone their routine vaccinations, posing a significant public health concern. While the impact has been global in nature, it is crucial to assess it at regional and country levels. For example, an analysis conducted in Africa estimated the prevalence of dropout rates for DPT vaccination, revealing Nigeria as the country with the highest recorded instances of immunization dropouts [13]. Similar evidence reports a drastic decline in childhood vaccination rates in the East Asia and Pacific region, with DTP3 coverage dropping by nine percentage points within just two years, reflecting the severity of the situation [13]. Studies conducted in the USA outlined a substantial impact on immunization across the country. They highlighted a notable decline (approximately 75%) in vaccines ordered by doctors since January when the first person with COVID-19 was identified. In the USA, the decline in childhood vaccination rates, as highlighted by the CDC, has been a matter of concern and appears to be closely tied to various factors associated with the implementation of social distancing measures during the ongoing COVID-19 pandemic. This decline included a notable reduction of over 50% in MCV for young children, as well as significant decreases in the uptake of other non-influenza vaccines [10].

However, regional variations in healthcare infrastructure, government responses, and cultural attitudes towards vaccination can significantly influence childhood vaccination coverage rates. Therefore, a nuanced evaluation on a regional and national scale is essential for understanding the full extent of the issue and implementing targeted strategies to address it effectively. For this reason, our study focused on the European region, covering a substantial time period from 2000 to 2022. We placed particular emphasis on the years 2019 to 2021, which were significantly impacted by the COVID-19 pandemic, and specifically examined childhood vaccination coverage for DTP and MCV.

For DTP1, there has been notable stability in coverage since 2000, with a consistent rate of 97% observed in 2022. Conversely, a slight increase of 1.1% was recorded for DTP3 between 2000 and 2022. However, both vaccines experienced a decrease of 1% in coverage during the COVID-19 pandemic. Shifting focus to MCV, the first-dose coverage was 91% in 2000, which increased to 93% by 2022. Similarly, the coverage for the second dose saw an upward trend from 89.6% to 91% during the same period. Nevertheless, the COVID-19 pandemic influenced these figures, reducing 2.1% for the first dose and 1% for the second dose. At the country level, the greatest impact of the pandemic was observed particularly in Eastern European countries. In some of these countries—such as Albania, Czechia, Latvia, Lithuania, the Republic of Moldova, Romania, and Slovakia—at least one joinpoint was identified in 2019 in the temporal trends of DTP and MCV coverage, reflecting a significant shift during the pandemic. It is also important to note that Hungary and Uzbekistan stood out in 2022 for achieving an impressive 99% coverage for all four essential vaccines. This milestone was previously attained by Hungary and Ukraine in 2000. However, while Hungary maintained this high coverage in 2022, Ukraine’s vaccination coverage for the same vaccines significantly declined between 2000 and 2022, with decreases of −26.3% and −21.2%. This shift in childhood vaccination coverage in Ukraine is likely attributable to the significant political events that have affected the country in recent years. The country faces endemic measles and increasing concerns over diphtheria prevalence, largely due to limited access to clean water, proper sanitation, and hygiene practices, which can exacerbate its spread. Moreover, suboptimal coverage of routine and childhood vaccinations compounds the situation. These factors underscore the urgent necessity for enhanced healthcare infrastructure and comprehensive vaccination initiatives to mitigate their impact [16].

To understand the fluctuating vaccination coverage of childhood vaccines in Europe during the study period, it is crucial to analyze the various factors that contributed to these changes. The identification of multiple joinpoints in the coverage rates for DTP1, MCV1, and MCV2 indicates that several elements—beyond the impact of the COVID-19 pandemic—have influenced vaccination trends. Unpacking these factors is key, particularly as data suggests that COVID-19 had no significant effect on MCV2 coverage, unlike other vaccines. An essential factor influencing vaccination uptake, both before and during the pandemic, is the role of parents in deciding whether their children receive immunizations [17,18]. Vaccine hesitancy is a significant factor contributing to fluctuating coverage rates, driven by misinformation, safety concerns, and cultural or religious beliefs. Misinformation, especially when propagated through social media and anti-vaccine movements, has undermined public confidence in immunization programs across Europe [17,18]. These hesitancy trends caused dips in vaccination rates as parents delayed or refused routine childhood vaccinations [17,18], contributing to several of the observed joinpoints in coverage data. During the pandemic, these challenges were exacerbated. Lockdowns, healthcare system disruptions, and concerns about exposure to the virus led many parents to avoid routine healthcare visits, resulting in missed vaccinations [19,20]. In addition to vaccine hesitancy, healthcare system disruptions also played a major role in fluctuating vaccination coverage, particularly during the COVID-19 pandemic [21,22,23]. National lockdowns, restrictions on movement, and the diversion of healthcare resources to pandemic management led to significant delays or missed routine childhood vaccinations [23]. This created gaps in coverage for vaccines like DTP1 and MCV1. Socioeconomic factors also played a key role in the observed fluctuations [24,25]. Lower vaccination coverage was often found in marginalized communities, rural areas, and populations with limited access to healthcare services [24,25]. Research consistently shows that disparities in healthcare access, vaccine availability, and public health outreach contribute to gaps in immunization coverage, especially in underserved populations [24,25,26]. Moreover, public trust in governmental and healthcare institutions is critical for maintaining stable vaccination rates [27,28]. Countries with high levels of trust in their health systems may tend to have more consistent coverage, while those with political instability or widespread distrust may experience more erratic fluctuations in vaccine uptake. Interestingly, despite these broad challenges, COVID-19 did not seem to have a significant impact on MCV2 coverage, which remained stable. It is important to note that MCV2 is administered at a later age, often as part of school-entry vaccination programs. Since many countries prioritized maintaining essential healthcare services for school-aged children during the pandemic, school-based immunization efforts may have been less disrupted compared to services for younger children [29,30].

To address gaps in immunity resulting from disruptions in immunization programs, it is crucial to implement comprehensive catch-up vaccination strategies alongside ongoing monitoring efforts [16]. Moreover, countries must tailor their immunization policies to tackle specific challenges and barriers to vaccination, including enhancing tracking systems to identify and follow up with individuals who are behind schedule for vaccines [31]. Training healthcare professionals to integrate catch-up vaccination strategies into their routine practices is vital for minimizing missed vaccination opportunities. Strategies should entail multifaceted initiatives to reach individuals who missed vaccine doses and ensure they receive the necessary immunizations. One pivotal aspect of catch-up vaccination strategies involves immediate interventions such as organizing mass vaccination campaigns. These campaigns aim to rapidly deliver vaccines to large populations, particularly targeting areas or communities with low immunization coverage [32]. In addition to healthcare settings, screening children for vaccination status during any encounter with the healthcare system or at school entry can help identify and address gaps in immunization coverage. This proactive approach ensures that children receive the necessary vaccines according to the recommended schedule. Moreover, expanding age-based eligibility for vaccinations ensures that older children and adolescents who missed vaccinations earlier in life could catch up on their immunizations [11].

Research indicates that facilitating access to vaccines has been instrumental in increasing vaccination coverage rates. This includes, apart from the above strategies, ensuring accessibility to immunization services, providing public coverage for vaccination costs, and conducting information and education campaigns. To ensure optimal protection against VPDs for all individuals, it is crucial to implement concrete crisis preparedness plans and take proactive measures to achieve and maintain robust and resilient vaccination systems [16].

Our study benefits from several strengths. These include the use of robust analytical methods such as joinpoint regression analysis, which allows for precise identification of significant changes in vaccination coverage trends over time. Additionally, our study’s comprehensive temporal coverage and focus on the effects of the COVID-19 pandemic provide valuable insights into the dynamics of childhood vaccination coverage in the WHO European Region. Furthermore, our methodological approach and consideration of multi-level analyses contribute to the reliability and validity of our findings, enhancing their applicability for informing public health policies and interventions at the regional and country level. However, our study has also some limitations. Firstly, relying on UNICEF databases for vaccination coverage data could introduce biases or inaccuracies due to variations in data collection methods and reporting systems across individual countries. These disparities in data quality and reporting standards may compromise the reliability and comparability of our findings. Secondly, while joinpoint regression analysis is a commonly used method for examining trends over time, it assumes linear relationships between variables within each segment. However, this assumption may not always be valid for complex phenomena like vaccination coverage, potentially hindering the model’s ability to accurately capture non-linear trends. Thirdly, although comparing AAPCs between pre-pandemic and pandemic periods offers insights into temporal variations, this approach may oversimplify the pandemic’s impact on vaccination coverage. To gain a comprehensive understanding of observed trends, it is crucial to consider additional contextual factors such as healthcare system disruptions, public health interventions, and vaccine hesitancy dynamics. Integrating these elements would enrich the analysis and provide a more nuanced interpretation of the data.

## 5. Conclusions

Our study highlights and quantifies the effect of the pandemic on childhood vaccination coverage. Furthermore, compared to a simple descriptive analysis, the temporal analysis of trends using joinpoint regression provides significant opportunities to identify potential factors driving variations in vaccination coverage levels and pinpoint areas for intervention in future vaccination strategies. By taking into account additional contextual factors such as disruptions in healthcare systems and dynamics of vaccine hesitancy, we may unlock significant potential for a more nuanced interpretation of the observed trends. Moving forward, our findings can inform evidence-based decision-making and facilitate targeted interventions to improve childhood vaccination coverage and mitigate the impact of future public health crises in the WHO European Region.

## Data Availability

The data presented in this study are available from UNICEF databases, reference number [13].

## Supplementary Materials

The following supporting information can be downloaded at: https://www.mdpi.com/article/10.3390/vaccines12101145/s1, Tables S1. Joinpoint regression analysis of DTP1 coverage at the country level. Tables S2. Joinpoint regression analysis of DTP3 coverage at the country level. Tables S3. Joinpoint regression analysis of MCV1 coverage at the country level. Tables S4. Joinpoint regression analysis of MCV2 coverage at the country level.

## References

[1] WHO (2020). Guiding Principles for Immunization Activities during the COVID-19 Pandemic.

[2] WHO (2020). At Least 80 Million Children under One at Risk of Diseases Such as Diphtheria, Measles and Polio as COVID-19 Disrupts Routine Vaccination Efforts, Warn Gavi, WHO and UNICEF.

[3] Moraga-Llop F.A., Fernández-Prada M., Grande-Tejada A.M., Martínez-Alcorta L.I., Moreno-Pérez D., Pérez-Martín J.J. (2020). Recovering lost vaccine coverage due to COVID-19 pandemic. Vacunas.

[4] ECDC ECDC Reports: Vaccine-Preventable Diseases on the Rise in the EU/EEA. https://www.bing.com/search?pglt=41&q=ECDC+reports%3A+vaccine-preventable+diseases+on+the+rise+in+the+EU%2FEEA&cvid=e31942add0794b8fba76ad0176e195b2&gs_lcrp=EgZjaHJvbWUyBggAEEUYOTIGCAEQRRg80gEHMzIwajBqMagCALACAA&FORM=ANNTA1&PC=DCTS.

[5] Shet A., Carr K., Danovaro-Holliday M.C., Sodha S.V., Prosperi C., Wunderlich J., Wonodi C., Reynolds H.W., Mirza I., Gacic-Dobo M. (2022). Impact of the SARS-CoV-2 pandemic on routine immunisation services: Evidence of disruption and recovery from 170 countries and territories. Lancet Glob. Health.

[6] UNICEF Over 13 Million Children Did Not Receive Any Vaccines at All Even Before COVID-19 Disrupted Global Immunization. https://www.unicef.org/press-releases/over-13-million-children-did-not-receive-any-vaccines-all-even-covid-19-disrupted.

[7] McDonald H.I., Tessier E., White J.M., Woodruff M., Knowles C., Bates C., Parry J., Walker J.L., Scott J.A., Smeeth L. (2020). Early impact of the coronavirus disease (COVID-19) pandemic and physical distancing measures on routine childhood vaccinations in England, January to April 2020. Eurosurveillance.

[8] Middeldorp M., van Lier A., van der Maas N., Veldhuijzen I., Freudenburg W., van Sorge N.M., Sanders E.A.M., Knol M.J., de Melker H.E. (2021). Short term impact of the COVID-19 pandemic on incidence of vaccine preventable diseases and participation in routine infant vaccinations in the Netherlands in the period March-September 2020. Vaccine.

[9] Akaba G., Dirisu O., Okunade K., Adams E., Ohioghame J., Obikeze O., Izuka E., Sulieman M., Edeh M. (2020). Impact of COVID-19 on utilization of maternal, newborn and child health services in Nigeria: Protocol for a country-level mixed-methods study. F1000Res.

[10] Nuzhath T., Ajayi K.V., Fan Q., Hotez P., Colwell B., Callaghan T., Regan A.K. (2021). Childhood immunization during the COVID-19 pandemic in Texas. Vaccine.

[11] Patel Murthy B., Zell E., Kirtland K., Jones-Jack N., Harris L., Sprague C., Schultz J., Le Q., Bramer C.A., Kuramoto S. (2021). Impact of the COVID-19 Pandemic on Administration of Selected Routine Childhood and Adolescent Vaccinations—10 U.S. Jurisdictions, March-September 2020. MMWR Morb. Mortal. Wkly. Rep..

[12] Bramer C.A., Kimmins L.M., Swanson R., Kuo J., Vranesich P., Jacques-Carroll L.A., Shen A.K. (2020). Decline in Child Vaccination Coverage During the COVID-19 Pandemic—Michigan Care Improvement Registry, May 2016–May 2020. MMWR Morb. Mortal. Wkly. Rep..

[13] Aguinaga-Ontoso I., Guillen-Aguinaga S., Guillen-Aguinaga L., Alas-Brun R., Onambele L., Aguinaga-Ontoso E., Guillen-Grima F. (2023). COVID-19 Impact on DTP Vaccination Trends in Africa: A Joinpoint Regression Analysis. Vaccines.

[14] UNICEF UNICEF Data Warehouse. https://data.unicef.org/resources/data_explorer/unicef_f/.

[15] Lerman P.M. (1980). Fitting Segmented Regression Models by Grid Search. J. R. Stat. Soc. Ser. C (Appl. Stat.).

[16] Alexander C., Cabrera M., Moore M., Lomazzi M. (2023). Driving Paediatric Vaccine Recovery in Europe. Vaccines.

[17] Abenova M., Shaltynov A., Jamedinova U., Semenova Y. (2023). Worldwide Child Routine Vaccination Hesitancy Rate among Parents of Children Aged 0–6 Years: A Systematic Review and Meta-Analysis of Cross-Sectional Studies. Vaccines.

[18] Yang X., Shi N., Liu C., Zhang J., Miao R., Jin H. (2024). Relationship between vaccine hesitancy and vaccination behaviors: Systematic review and meta-analysis of observational studies. Vaccine.

[19] Alharbi H.S. (2023). Review: Factors influencing parents’ decisions to vaccinate children against COVID-19. Vaccine.

[20] Chan P.S., Fang Y., Kawuki J., Chen S., Liang X., Mo P.K., Wang Z. (2023). Parental Acceptance, Parental Hesitancy, and Uptake of Seasonal Influenza Vaccination among Children Aged 6-59 Months: A Systematic Review and Meta-Analysis. Vaccines.

[21] Shmueli M., Lendner I., Ben-Shimol S. (2024). Effect of the COVID-19 pandemic on the pediatric infectious disease landscape. Eur. J. Pediatr..

[22] Cardoso Pinto A.M., Shariq S., Ranasinghe L., Sundar Budhathoki S., Skirrow H., Whittaker E., Seddon J.A. (2023). Reasons for reductions in routine childhood immunisation uptake during the COVID-19 pandemic in low- and middle-income countries: A systematic review. PLOS Glob. Public Health.

[23] Billon-Denis E., Tournier J.N. (2020). COVID-19 and vaccination: A global disruption. Med. Sci..

[24] Pereira M.A.D., Arroyo L.H., Gallardo M., Arcêncio R.A., Gusmão J.D., Amaral G.G., Oliveira V.C., Guimarães E.A.A. (2023). Vaccination coverage in children under one year of age and associated socioeconomic factors: Maps of spatial heterogeneity. Rev. Bras. Enferm..

[25] de Figueiredo A., Johnston I.G., Smith D.M., Agarwal S., Larson H.J., Jones N.S. (2016). Forecasted trends in vaccination coverage and correlations with socioeconomic factors: A global time-series analysis over 30 years. Lancet Glob. Health.

[26] Lanke R., Chimurkar V. (2024). Measles Outbreak in Socioeconomically Diverse Sections: A Review. Cureus.

[27] Bolsewicz K.T., Steffens M.S., King C., Abdi I., Bullivant B., Beard F. (2023). A qualitative study on COVID-19 pandemic impacts on parental attitudes and intentions for routine adolescent vaccinations: The role of trust. Vaccine.

[28] (2022). Pandemic preparedness and COVID-19: An exploratory analysis of infection and fatality rates, and contextual factors associated with preparedness in 177 countries, from Jan 1, 2020, to Sept 30, 2021. Lancet.

[29] Sahoo K.C., Negi S., Patel K., Mishra B.K., Palo S.K., Pati S. (2021). Challenges in Maternal and Child Health Services Delivery and Access during Pandemics or Public Health Disasters in Low-and Middle-Income Countries: A Systematic Review. Healthcare.

[30] Ota M.O.C., Badur S., Romano-Mazzotti L., Friedland L.R. (2021). Impact of COVID-19 pandemic on routine immunization. Ann. Med..

[31] (2021). Measuring routine childhood vaccination coverage in 204 countries and territories, 1980–2019: A systematic analysis for the Global Burden of Disease Study 2020, Release 1. Lancet.

[32] Muhoza P., Danovaro-Holliday M.C., Diallo M.S., Murphy P., Sodha S.V., Requejo J.H., Wallace A.S. (2021). Routine Vaccination Coverage—Worldwide, 2020. MMWR Morb. Mortal. Wkly. Rep..

