# Supplementary material for: Trends of Diphtheria–Tetanus–Pertussis and Measles Vaccine Coverage Preceding and during the COVID-19 Pandemic: An Analysis of the WHO European Region from 2000 to 2022
Source: Vaccines (Basel). 2024 Oct 6;12(10):1145. doi: 10.3390/vaccines12101145 (PMC11511252; doi:10.3390/vaccines12101145)
Supplement: Supplementary file 1 [file vaccines-12-01145-s001.zip › vaccines-3236766-supplementary.pdf]

SUPPLEMENTARY MATERIALS

Tables S1. Joinpoint regression analysis of DTP1 coverage at the country level

Geographic area	Joinpoint segment	APC	APC 95% LCI	APC 95% UCI	p-value
Albania	2000-2013	0.14	0.07	0.22	<0.001
	2013-2022	-0.11	-0.23	0.01	0.081
Andorra	2000-2022	0.05	-0.02	0.13	0.173
Armenia	2000-2003	0.29	-0.23	0.82	0.248
	2003-2006	-1.10	-2.13	-0.07	0.039
	2006-2010	0.78	0.25	1.30	0.007
	2010-2022	-0.19	-0.25	-0.13	<0.001
Austria	2000-2022	-0.15	-0.39	0.09	0.220
Azerbaijan	2000-2016	1.27	0.96	1.59	<0.001
	2016-2022	-1.76	-3.10	-0.40	0.014
Belarus	2000-2022	0.16	-0.23	0.56	0.403
Belgium	2000-2003	-0.11	-0.40	0.18	0.418
	2003-2006	0.74	0.15	1.33	0.018
	2006-2016	0.02	-0.04	0.07	0.516
	2016-2022	-0.22	-0.31	-0.12	<0.001
Bosnia and Herzegovina	2000-2010	0.63	0.13	1.13	0.017
	2010-2022	-1.09	-1.47	-0.72	<0.001
Bulgaria	2000-2022	-0.13	-0.24	-0.03	0.017
Croatia	2000-2022	0.12	0.05	0.18	0.001
Cyprus	2000-2012	0.10	0.03	0.16	0.006
	2012-2022	-0.10	-0.19	-0.02	0.023
Czechia	2000-2020	-0.02	-0.04	0.00	0.112
	2020-2022	-0.43	-1.30	0.44	0.311
Denmark	2000-2003	0.27	-1.07	1.63	0.669
	2003-2007	-2.82	-4.12	-1.51	0.001
	2007-2012	1.86	1.00	2.73	<0.001
	2012-2022	0.17	-0.04	0.38	0.100
Estonia	2000-2002	1.62	0.11	3.15	0.037
	2002-2020	-0.41	-0.46	-0.36	<0.001
	2020-2022	-3.18	-4.62	-1.72	<0.001
Finland	2000-2015	0.03	-0.02	0.08	0.278
	2015-2022	-0.24	-0.39	-0.09	0.004
France	2000-2022	0.00	0.00	0.00	0.014
Georgia	2000-2022	0.20	-0.02	0.41	0.070
Germany	2000-2008	0.40	0.31	0.49	<0.001
	2008-2015	-0.20	-0.33	-0.07	0.006
	2015-2022	0.01	-0.10	0.11	0.913
Greece	2000-2004	-0.06	-0.06	-0.06	<0.001
	2004-2007	1.12	1.12	1.12	<0.001
	2007-2022	-0.01	-0.01	-0.01	<0.001
Hungary	2000-2022	0.00	0.00	0.00	0.014
Iceland	2000-2022	-0.07	-0.16	0.02	0.144
Ireland	2000-2002	-0.19	-1.08	0.72	0.664
	2002-2009	0.56	0.40	0.71	<0.001

	2009-2022	-0.03	-0.08	0.02	0.212
Israel	2000-2012	-0.05	-0.25	0.16	0.631
	2012-2022	0.56	0.29	0.83	<0.001
Italy	2000-2022	-0.09	-0.17	-0.01	0.024
Kazakhstan	2000-2022	0.09	-0.14	0.32	0.428
Kyrgyzstan	2000-2019	-0.15	-0.26	-0.04	0.011
	2019-2022	-3.02	-4.81	-1.19	0.003
Latvia	2000-2022	-0.06	-0.20	0.09	0.429
Lithuania	2000-2022	-0.18	-0.27	-0.08	0.001
Luxembourg	2000-2022	0.00	0.00	0.00	0.014
Malta	2000-2007	-2.17	-2.89	-1.44	<0.001
	2007-2011	3.65	0.80	6.57	0.015
	2011-2022	-0.03	-0.40	0.35	0.881
Monaco	2000-2022	0.00	0.00	0.00	0.014
Montenegro	2006-2012	0.31	-0.18	0.80	0.195
	2012-2022	-0.54	-0.76	-0.32	<0.001
Netherlands (Kingdom of the)	2000-2022	-0.07	-0.10	-0.04	<0.001
North Macedonia	2000-2018	0.05	-0.12	0.22	0.571
	2018-2022	-2.01	-3.64	-0.36	0.020
Norway	2000-2018	0.00	-0.01	0.01	0.999
	2018-2022	-0.30	-0.43	-0.18	<0.001
Poland	2000-2022	0.00	0.00	0.00	0.014
Portugal	2000-2022	0.05	-0.03	0.13	0.184
Republic of Moldova	2000-2022	-0.50	-0.69	-0.30	<0.001
Romania	2000-2022	-0.25	-0.35	-0.15	<0.001
Russian Federation	2000-2008	0.35	0.22	0.48	<0.001
	2008-2011	-0.80	-1.94	0.36	0.163
	2011-2022	0.01	-0.07	0.09	0.759
San Marino	2000-2014	-0.79	-1.07	-0.51	<0.001
	2014-2022	0.62	-0.04	1.29	0.064
Serbia	2000-2022	0.11	-0.03	0.25	0.110
Slovakia	2000-2019	0.00	-0.01	0.00	0.368
	2019-2022	-0.78	-0.92	-0.63	<0.001
Slovenia	2000-2006	0.53	0.30	0.77	<0.001
	2006-2020	-0.11	-0.18	-0.04	0.005
	2020-2022	-3.30	-4.61	-1.98	<0.001
Spain	2000-2022	-0.02	-0.05	0.01	0.135
Sweden	2000-2018	0.00	-0.03	0.03	0.990
	2018-2022	-0.61	-0.86	-0.36	<0.001
Switzerland	2000-2022	0.17	0.12	0.22	<0.001
Tajikistan	2000-2004	1.51	0.79	2.25	0.001
	2004-2007	-1.54	-3.75	0.72	0.162
	2007-2010	2.79	0.48	5.15	0.021
	2010-2022	0.09	-0.05	0.22	0.185
Turkmenistan	2000-2022	0.09	0.04	0.14	0.002
Ukraine	2000-2016	-4.10	-5.81	-2.35	<0.001
	2016-2022	10.02	1.62	19.11	0.021

United Kingdom	2000-2008	-0.01	-0.07	0.06	0.789
	2008-2011	0.38	-0.20	0.96	0.178
	2011-2017	-0.02	-0.15	0.11	0.701
	2017-2022	-0.24	-0.37	-0.11	0.002
Uzbekistan	2000-2022	-0.03	-0.10	0.04	0.343

Tables S2. Joinpoint regression analysis of DTP3 coverage at the country level

Geographic area	Joinpoint segment	APC	APC 95% LCI	APC 95% UCI	p-value
Albania	2000-2018	0.12	0.06	0.17	<0.001
	2018-2022	-0.51	-1.04	0.03	0.064
Andorra	2000-2022	0.07	-0.03	0.17	0.151
Armenia	2000-2003	0.27	-1.24	1.81	0.704
	2003-2006	-2.62	-5.54	0.38	0.081
	2006-2011	1.83	0.86	2.82	0.001
	2011-2022	-0.32	-0.53	-0.12	0.005
Austria	2000-2009	0.12	-0.53	0.77	0.704
	2009-2013	3.18	-0.42	6.90	0.080
	2013-2022	-1.54	-2.17	-0.90	<0.001
Azerbaijan	2000-2006	0.00	-1.75	1.78	0.999
	2006-2016	2.54	1.56	3.52	<0.001
	2016-2022	-2.82	-4.52	-1.08	0.004
Belarus	2000-2022	0.48	-0.43	1.41	0.285
Belgium	2000-2003	-0.14	-0.86	0.59	0.674
	2003-2006	1.12	-0.34	2.60	0.117
	2006-2014	0.14	-0.06	0.33	0.148
	2014-2020	-0.33	-0.65	0.00	0.048
	2020-2022	0.59	-0.86	2.06	0.383
Bosnia and Herzegovina	2000-2012	0.61	-0.06	1.29	0.073
	2012-2018	-3.89	-6.30	-1.42	0.005
	2018-2022	0.94	-2.62	4.63	0.588
Bulgaria	2000-2022	-0.22	-0.34	-0.10	0.001
Croatia	2000-2005	0.61	0.22	1.00	0.007
	2005-2013	0.00	-0.23	0.23	0.995
	2013-2017	-1.04	-1.90	-0.18	0.024
	2017-2020	0.68	-1.06	2.45	0.403
	2020-2022	-1.31	-3.02	0.42	0.120
Cyprus	2000-2012	0.16	0.01	0.30	0.038
	2012-2022	-0.32	-0.51	-0.12	0.003
Czechia	2000-2012	0.13	0.01	0.25	0.038
	2012-2016	-0.82	-1.83	0.20	0.105
	2016-2019	0.51	-1.52	2.58	0.598
	2019-2022	-1.25	-2.26	-0.24	0.020
Denmark	2000-2008	-1.28	-1.81	-0.74	<0.001
	2008-2022	0.74	0.50	0.97	<0.001
Estonia	2000-2006	0.39	0.09	0.69	0.015
	2006-2020	-0.29	-0.38	-0.20	<0.001
	2020-2022	-3.27	-4.98	-1.54	0.001
Finland	2000-2014	0.06	-0.06	0.17	0.324
	2014-2017	-3.08	-5.45	-0.65	0.017
	2017-2022	0.11	-0.44	0.67	0.678
France	2000-2007	0.31	0.17	0.46	0.001
	2007-2013	0.03	-0.22	0.28	0.807
	2013-2016	-1.00	-2.10	0.11	0.073

	2016-2022	0.00	-0.19	0.19	0.978
Georgia	2000-2004	-2.09	-5.63	1.59	0.242
	2004-2007	6.90	-4.87	20.12	0.241
	2007-2022	-0.44	-0.93	0.05	0.073
Germany	2000-2003	2.18	1.31	3.07	<0.001
	2003-2010	-0.05	-0.34	0.24	0.729
	2010-2017	-0.76	-1.05	-0.47	<0.001
	2017-2022	0.03	-0.35	0.42	0.866
Greece	2000-2003	1.71	1.47	1.96	<0.001
	2003-2007	1.36	1.12	1.60	<0.001
	2007-2022	0.00	-0.02	0.02	0.940
Hungary	2000-2022	0.00	0.00	0.00	0.014
Iceland	2000-2009	0.27	-0.21	0.75	0.248
	2009-2012	-2.57	-7.53	2.66	0.306
	2012-2022	0.19	-0.21	0.60	0.326
Ireland	2000-2002	-1.48	-2.68	-0.27	0.021
	2002-2005	2.93	1.68	4.20	<0.001
	2005-2013	0.71	0.55	0.87	<0.001
	2013-2022	-0.33	-0.44	-0.22	<0.001
Israel	2000-2022	0.29	0.20	0.37	<0.001
Italy	2000-2003	2.87	0.98	4.79	0.005
	2003-2022	-0.08	-0.19	0.03	0.123
Kazakhstan	2000-2022	-0.02	-0.39	0.36	0.924
Kyrgyzstan	2000-2022	-0.37	-0.53	-0.21	<0.001
Latvia	2000-2004	0.73	-0.78	2.26	0.315
	2004-2011	-1.17	-1.97	-0.37	0.008
	2011-2019	1.05	0.41	1.70	0.004
	2019-2022	-1.58	-3.90	0.80	0.172
Lithuania	2000-2006	-0.12	-0.40	0.17	0.383
	2006-2009	1.11	-0.58	2.82	0.172
	2009-2012	-1.78	-3.42	-0.12	0.039
	2012-2016	0.54	-0.30	1.39	0.183
	2016-2022	-0.78	-1.07	-0.50	<0.001
Luxembourg	2000-2022	0.01	-0.01	0.02	0.236
Malta	2000-2005	-0.77	-2.56	1.05	0.373
	2005-2008	-9.17	-16.28	-1.45	0.025
	2008-2012	9.47	5.10	14.03	<0.001
	2012-2022	-0.07	-0.70	0.57	0.816
Monaco	2000-2022	0.00	0.00	0.00	0.014
Montenegro	2006-2011	0.98	0.16	1.80	0.023
	2011-2022	-1.47	-1.71	-1.23	<0.001
Netherlands (Kingdom of the)	2000-2011	-0.03	-0.18	0.13	0.737
	2011-2022	-0.36	-0.52	-0.20	<0.001
North Macedonia	2000-2016	0.03	-0.25	0.31	0.837
	2016-2022	-2.44	-3.64	-1.23	0.001
Norway	2000-2022	0.30	0.25	0.36	<0.001
Poland	2000-2015	0.02	-0.08	0.12	0.744

	2015-2022	-1.45	-1.76	-1.14	<0.001
Portugal	2000-2022	0.14	0.06	0.22	0.002
Republic of Moldova	2000-2022	-0.51	-0.70	-0.31	<0.001
Romania	2000-2022	-0.76	-0.90	-0.61	<0.001
Russian Federation	2000-2007	0.36	0.23	0.49	<0.001
	2007-2011	-0.32	-0.80	0.16	0.180
	2011-2022	0.00	-0.06	0.07	0.980
San Marino	2000-2015	-0.93	-1.12	-0.75	<0.001
	2015-2022	0.98	0.38	1.58	0.003
Serbia	2000-2022	0.02	-0.15	0.20	0.792
Slovakia	2000-2012	0.00	-0.03	0.03	0.889
	2012-2015	-1.07	-1.59	-0.55	0.001
	2015-2022	0.19	0.12	0.26	<0.001
Slovenia	2000-2008	0.85	0.22	1.48	0.011
	2008-2022	-0.54	-0.81	-0.28	<0.001
Spain	2000-2016	0.01	-0.10	0.11	0.921
	2016-2022	-0.75	-1.22	-0.28	0.004
Sweden	2000-2022	-0.12	-0.17	-0.06	<0.001
Switzerland	2000-2003	-0.01	-0.34	0.32	0.925
	2003-2012	0.36	0.29	0.43	<0.001
	2012-2022	-0.01	-0.06	0.05	0.835
Tajikistan	2000-2006	-0.13	-0.76	0.50	0.664
	2006-2011	2.59	1.39	3.81	<0.001
	2011-2022	0.15	-0.10	0.40	0.217
Turkmenistan	2000-2022	0.20	-0.02	0.42	0.073
Ukraine	2000-2012	-4.11	-8.43	0.41	0.071
	2012-2015	-27.99	-66.98	57.03	0.384
	2015-2022	23.23	11.04	36.76	0.001
United Kingdom	2000-2008	0.18	0.06	0.30	0.006
	2008-2012	0.91	0.37	1.46	0.003
	2012-2022	-0.34	-0.42	-0.25	<0.001
Uzbekistan	2000-2007	-0.41	-0.72	-0.09	0.019
	2007-2010	0.76	-1.63	3.21	0.492
	2010-2017	0.00	-0.40	0.41	0.986
	2017-2020	-1.31	-3.65	1.08	0.244
	2020-2022	2.19	-0.23	4.67	0.072

Tables S3. Joinpoint regression analysis of MCV1 coverage at the country level

Geographic area	Joinpoint segment	APC	APC 95% LCI	APC 95% UCI	p-value
Albania	2000-2013	0.36	0.19	0.53	<0.001
	2013-2019	-0.86	-1.57	-0.14	0.022
	2019-2022	-3.17	-4.72	-1.60	0.001
Andorra	2000-2006	-0.90	-1.55	-0.25	0.011
	2006-2010	1.73	-0.24	3.74	0.080
	2010-2013	-1.30	-5.08	2.63	0.480
	2013-2022	0.37	0.01	0.73	0.045
Armenia	2000-2007	0.02	-0.35	0.39	0.928
	2007-2010	1.81	-0.97	4.67	0.187
	2010-2022	-0.29	-0.45	-0.12	0.002
Austria	2000-2010	0.44	-0.23	1.11	0.181
	2010-2014	4.68	0.30	9.24	0.037
	2014-2022	0.00	-0.92	0.94	0.994
Azerbaijan	2000-2005	0.12	-2.42	2.73	0.921
	2005-2013	5.15	3.54	6.78	<0.001
	2013-2022	-1.20	-2.23	-0.16	0.027
Belarus	2000-2022	-0.05	-0.10	-0.01	0.024
Belgium	2000-2003	-0.51	-0.94	-0.08	0.025
	2003-2006	4.01	3.11	4.92	<0.001
	2006-2010	1.17	0.73	1.61	<0.001
	2010-2022	0.06	0.01	0.11	0.034
Bosnia and Herzegovina	2000-2012	0.71	-0.20	1.62	0.118
	2012-2022	-4.89	-6.01	-3.75	<0.001
Bulgaria	2000-2005	1.78	0.67	2.90	0.003
	2005-2022	-0.43	-0.60	-0.26	<0.001
Croatia	2000-2008	0.36	-0.08	0.81	0.105
	2008-2022	-0.52	-0.71	-0.33	<0.001
Cyprus	2000-2013	0.03	-0.11	0.17	0.625
	2013-2016	1.43	-1.19	4.12	0.267
	2016-2022	-0.97	-1.41	-0.53	<0.001
Czechia	2000-2016	0.13	0.06	0.19	0.001
	2016-2019	-2.04	-3.70	-0.35	0.021
	2019-2022	1.65	0.78	2.52	0.001
Denmark	2000-2005	-0.55	-1.50	0.41	0.234
	2005-2009	-2.85	-4.91	-0.75	0.012
	2009-2017	1.61	1.03	2.20	<0.001
	2017-2022	-0.16	-1.11	0.80	0.724
Estonia	2000-2015	-0.09	-0.34	0.15	0.427
	2015-2022	-1.41	-2.17	-0.64	0.001
Finland	2000-2010	0.23	0.09	0.36	0.003
	2010-2016	-0.65	-1.04	-0.27	0.003
	2016-2019	0.52	-1.19	2.26	0.522
	2019-2022	-0.89	-1.74	-0.04	0.042
France	2000-2006	0.91	0.49	1.33	<0.001
	2006-2018	0.17	0.00	0.34	0.046

	2018-2022	1.09	0.31	1.88	0.009
Georgia	2000-2006	4.92	2.61	7.29	<0.001
	2006-2022	-0.02	-0.52	0.49	0.951
Germany	2000-2004	0.91	0.66	1.16	<0.001
	2004-2008	0.29	-0.09	0.68	0.128
	2008-2022	0.00	-0.04	0.04	0.962
Greece	2000-2007	1.49	1.38	1.59	<0.001
	2007-2012	-0.02	-0.26	0.22	0.852
	2012-2015	-0.72	-1.47	0.03	0.058
	2015-2022	0.02	-0.08	0.12	0.722
Hungary	2000-2022	0.00	0.00	0.00	0.014
Iceland	2000-2022	0.02	-0.11	0.16	0.738
Ireland	2000-2002	-3.29	-5.10	-1.44	0.002
	2002-2005	5.38	3.41	7.39	<0.001
	2005-2013	1.18	0.92	1.43	<0.001
	2013-2022	-0.40	-0.57	-0.23	<0.001
Israel	2000-2022	0.19	0.13	0.24	<0.001
Italy	2000-2004	4.11	3.11	5.12	<0.001
	2004-2012	0.59	0.17	1.00	0.010
	2012-2015	-2.32	-5.26	0.72	0.117
	2015-2018	3.15	0.04	6.36	0.047
	2018-2022	0.06	-0.90	1.04	0.885
Kazakhstan	2000-2022	0.00	-0.12	0.12	0.981
Kyrgyzstan	2000-2015	-0.07	-0.21	0.07	0.335
	2015-2022	-0.77	-1.22	-0.33	0.002
Latvia	2000-2012	-0.50	-0.79	-0.21	0.002
	2012-2022	0.57	0.18	0.96	0.006
Lithuania	2000-2008	-0.03	-0.29	0.23	0.809
	2008-2012	-1.11	-2.28	0.07	0.062
	2012-2017	0.27	-0.48	1.02	0.453
	2017-2022	-1.56	-2.08	-1.03	<0.001
Luxembourg	2000-2010	0.27	0.21	0.34	<0.001
	2010-2013	0.96	0.12	1.81	0.027
	2013-2022	-0.01	-0.09	0.07	0.780
Malta	2000-2022	1.14	0.54	1.75	0.001
Monaco	2000-2017	-0.65	-0.71	-0.60	<0.001
	2017-2022	-0.05	-0.41	0.31	0.771
Montenegro	2006-2013	-0.46	-7.75	7.40	0.897
	2013-2022	-14.35	-18.69	-9.79	<0.001
Netherlands (Kingdom of the)	2000-2014	0.05	-0.01	0.11	0.091
	2014-2017	-1.16	-2.38	0.08	0.064
	2017-2020	0.58	-0.66	1.84	0.327
	2020-2022	-2.66	-3.86	-1.44	<0.001
North Macedonia	2000-2013	0.19	-0.13	0.51	0.224
	2013-2020	-5.10	-6.07	-4.11	<0.001
	2020-2022	2.66	-3.45	9.15	0.377
Norway	2000-2003	-0.89	-2.19	0.42	0.167

	2003-2006	1.97	-0.68	4.69	0.135
	2006-2022	0.36	0.25	0.46	<0.001
Poland	2000-2018	-0.12	-0.32	0.08	0.212
	2018-2022	-8.05	-9.86	-6.22	<0.001
Portugal	2000-2003	2.87	1.41	4.36	0.001
	2003-2022	0.22	0.14	0.31	<0.001
Republic of Moldova	2000-2019	-0.21	-0.46	0.05	0.108
	2019-2022	-3.60	-7.67	0.66	0.092
Romania	2000-2012	-0.39	-0.53	-0.24	<0.001
	2012-2016	-2.48	-3.70	-1.24	0.001
	2016-2019	2.05	-0.49	4.65	0.105
	2019-2022	-2.75	-3.97	-1.52	<0.001
Russian Federation	2000-2006	0.39	0.24	0.53	<0.001
	2006-2010	-0.30	-0.72	0.13	0.155
	2010-2018	0.00	-0.11	0.11	0.989
	2018-2022	-0.31	-0.58	-0.04	0.029
San Marino	2000-2014	-1.29	-1.56	-1.01	<0.001
	2014-2022	1.28	0.63	1.94	0.001
Serbia	2000-2009	0.69	-0.47	1.86	0.231
	2009-2022	-1.32	-1.97	-0.66	0.001
Slovakia	2000-2012	0.00	-0.10	0.10	0.981
	2012-2015	-1.02	-2.66	0.64	0.207
	2015-2022	-0.03	-0.25	0.19	0.770
Slovenia	2000-2002	-0.97	-3.02	1.12	0.328
	2002-2008	0.55	0.08	1.02	0.026
	2008-2017	-0.43	-0.65	-0.20	0.002
	2017-2022	0.66	0.19	1.13	0.010
Spain	2000-2022	0.00	-0.08	0.08	0.955
Sweden	2000-2002	2.42	0.60	4.27	0.012
	2002-2020	0.12	0.05	0.18	0.001
	2020-2022	-2.56	-4.28	-0.80	0.008
Switzerland	2000-2004	0.26	-0.23	0.76	0.272
	2004-2008	2.58	1.78	3.38	<0.001
	2008-2022	0.37	0.30	0.44	<0.001
Tajikistan	2000-2003	1.84	-0.61	4.36	0.128
	2003-2006	-3.69	-8.27	1.13	0.119
	2006-2011	3.04	1.46	4.64	0.001
	2011-2022	0.27	-0.06	0.60	0.101
Turkmenistan	2000-2022	0.14	-0.02	0.29	0.079
Ukraine	2000-2015	-3.86	-6.05	-1.63	0.002
	2015-2022	6.10	-1.35	14.10	0.105
United Kingdom	2000-2004	-1.88	-2.66	-1.08	<0.001
	2004-2013	1.55	1.26	1.83	<0.001
	2013-2022	-0.33	-0.56	-0.10	0.008
Uzbekistan	2000-2022	0.01	-0.06	0.09	0.722

Tables S4. Joinpoint regression analysis of MCV2 coverage at the country level

Geographic area	Joinpoint segment	APC	APC 95% LCI	APC 95% UCI	p-value
Armenia	2000-2006	-1.26	-1.69	-0.83	<0.001
	2006-2010	2.39	1.09	3.71	0.001
	2010-2022	-0.34	-0.49	-0.18	<0.001
Austria	2000-2012	7.21	6.17	8.26	<0.001
	2012-2022	1.35	0.05	2.67	0.042
Belarus	2000-2002	2.89	-0.08	5.94	0.056
	2002-2022	0.00	-0.08	0.08	0.982
Bosnia and Herzegovina	2000-2002	8.68	-3.47	22.36	0.155
	2002-2014	0.25	-0.55	1.05	0.519
	2014-2022	-5.59	-6.80	-4.36	<0.001
Bulgaria	2000-2007	2.26	0.89	3.64	0.003
	2007-2022	-0.65	-1.08	-0.23	0.005
Czechia	2000-2015	0.01	-0.09	0.11	0.872
	2015-2018	-4.55	-6.84	-2.21	0.001
	2018-2022	1.69	0.91	2.47	<0.001
Estonia	2000-2004	3.41	1.45	5.40	0.002
	2004-2020	-0.62	-0.88	-0.37	<0.001
	2020-2022	-11.31	-16.51	-5.78	0.001
Hungary	2000-2022	0.00	0.00	0.00	0.014
Kazakhstan	2000-2022	-0.07	-0.20	0.06	0.275
Kyrgyzstan	2000-2022	-0.09	-0.17	0.00	0.042
Latvia	2000-2004	2.02	-0.10	4.19	0.060
	2004-2016	-0.94	-1.38	-0.49	0.001
	2016-2019	2.72	-3.88	9.78	0.395
	2019-2022	-4.13	-7.26	-0.90	0.017
Lithuania	2000-2003	-1.43	-2.56	-0.29	0.020
	2003-2008	0.57	-0.16	1.30	0.113
	2008-2014	-0.73	-1.24	-0.22	0.010
	2014-2019	0.19	-0.53	0.93	0.560
	2019-2022	-2.16	-3.28	-1.03	0.002
North Macedonia	2000-2022	-0.57	-1.04	-0.10	0.019
Norway	2000-2004	-0.13	-1.10	0.85	0.776
	2004-2010	1.30	0.60	2.00	0.002
	2010-2013	-2.31	-5.28	0.76	0.126
	2013-2022	0.60	0.32	0.88	0.001
Poland	2000-2022	-0.15	-0.28	-0.02	0.029
Portugal	2000-2002	16.08	14.25	17.93	<0.001
	2002-2006	10.15	9.28	11.03	<0.001
	2006-2022	0.01	-0.05	0.07	0.686
Romania	2000-2011	-0.42	-0.81	-0.02	0.042
	2011-2016	-3.54	-5.33	-1.71	0.001
	2016-2022	-1.01	-2.00	-0.01	0.048
Russian Federation	2000-2003	4.35	3.60	5.11	<0.001
	2003-2022	-0.01	-0.05	0.03	0.662
Serbia	2000-2004	6.62	1.49	12.02	0.014

	2004-2022	-0.50	-0.99	0.01	0.052
Slovakia	2000-2013	0.01	-0.06	0.09	0.672
	2013-2016	-0.64	-2.04	0.77	0.339
	2016-2020	0.20	-0.51	0.91	0.553
	2020-2022	-1.08	-2.47	0.33	0.121
Slovenia	2000-2005	0.28	-0.29	0.85	0.312
	2005-2022	-0.47	-0.56	-0.38	<0.001
Sweden	2000-2017	0.04	-0.01	0.09	0.079
	2017-2022	-0.98	-1.26	-0.69	<0.001
Tajikistan	2000-2002	25.82	15.45	37.12	<0.001
	2002-2022	0.26	0.02	0.49	0.035
Turkmenistan	2000-2003	-1.81	-3.42	-0.17	0.033
	2003-2006	4.07	0.68	7.58	0.021
	2006-2022	-0.03	-0.15	0.10	0.673
Ukraine	2000-2014	-5.65	-8.88	-2.31	0.003
	2014-2022	7.43	-0.93	16.51	0.080
United Kingdom	2000-2008	0.03	-0.37	0.43	0.887
	2008-2012	4.21	2.32	6.14	<0.001
	2012-2022	-0.21	-0.50	0.07	0.134
Uzbekistan	2000-2005	-0.95	-2.31	0.43	0.158
	2005-2009	-36.23	-38.17	-34.22	<0.001
	2009-2012	95.62	83.90	108.08	<0.001
	2012-2022	-2.36	-2.83	-1.89	<0.001
